# The Statistical Analysis of Top Hubs in Growing Geographical Networks with Optimal Policy

**DOI:** 10.1038/s41598-019-45783-y

**Published:** 2019-06-26

**Authors:** Li-Sheng Zhang, Chun-Lei Li

**Affiliations:** School of Science, North China University of Technology, Beijing, 100144 P.R. China

**Keywords:** Complex networks, Statistical physics

## Abstract

Many practical networks, such as city networks, road networks and neural networks, usually grow up on basis of topological structures and geographical measures. Big hubs, importance of which have been well known in complex networks, still play crucial roles in growing networks with geographical measures. Therefore, it is very necessary to investigate the underlying mechanisms of statistical features of different top hubs in such networks. Here, we propose a growing network model based on optimal policy in geographical ground. Through the statistics of a great number of geographical networks, we find that the degree and position distributions of top four hubs are diverse between them and closely interrelated with each other, and further gain the relationships between the upper limits of top hubs and the size of networks. Then, the underlying mechanisms are explored. Meanwhile, we are diligent to obtain the corresponding relationships of different spatial distribution areas for different top hubs, and compute their abnormal average degrees at different spatial positions, which show significant differences and imply the advantage of spatial positions and intense competition between top hubs. We hope our results could offer useful inspirations for related practical network studies.

## Introduction

With the development of network science, studies of complex networks have extended to diverse disciplines^1–3^, such as physics^4–6^, mathematics and control theory^7–9^, social systems^10,11^ and so on. Different growing network models are proposed to investigate special practical problems^12,13^. For many real systems, e.g., road networks^14^, flight networks^15^, power grids^16^ and so on, spatial constraint must be considered. Therefore, growing geographical networks have become an object of extensive investigation^17–19^ for recent years, such as gravity models in literatures^20,21^. In fact, there have made extraordinary progress in the studies of geographical networks. Karpiarz *et al*. find that the distance scaling coefficient is strictly related to the fractal dimension of international trade network, which help them cope with the globalization puzzle from gravity model of trade^22^. In order to evaluate the spatial effects in the real networks, Ruzzenenti *et al*. propose an effective solution by introducing global and local measures of spatial effect through a comparison with adequate null models^23^. Masuda *et al*. use geographical nongrowing network model and find the small-world networks with power-law degree distributions in appropriate configurations^24^. Yakubo *et al*. successfully explain both superlinear and sublinear allometric scaling of urban indicators that quantify activities or performances of the city by using a geographical scale-free network to describe relations between people in a city^19^. Moreover, lots of network science studies^1^ have suggested that big hubs are frequently important (but not always) for both topological structures and dynamical functions in whole networks^25–27^. So the features of big hubs are usually paid more attention from researchers^28–30^. Although existing results are rich and outstanding in previous studies, there is little discussion on distribution relationships between top hubs in growing geographical networks. Therefore, it is much significant to explore the different features of degree and position distributions for different top hubs in growing geographical networks.

In this paper, it is proposed that a growing network model embedded in geographical space. Through a great number of networks, we find the different characteristics of degree distributions and spatial distributions for top hubs, get the variation of the upper limits of top hubs with network size. And the underlying mechanisms of such results are studied. As a result of competitions between big hubs in geographical space, the corresponding relationships of spatial distribution areas of top four hubs are obtained. Moreover, we analyze the effects of attraction of top hubs and the spatial position advantage on the average degrees over spatial positions, and gain the reason of heterogenous average degree in geographical space.

Our analysis is set out as follows. In the next section, it is designed and applied in the whole paper that the growing network model based on optimal policies involving both topological and geographical measures. Then, we numerically simulate growing behaviors of networks, analyze the statistical features of top hubs in generated networks and uncover underlying mechanisms in Sec.III. At last, we conclude these results and possible significance for applications.

## Network Model

The present model is designed in one dimensional space. During the growth of geographical networks, the position of any node is random. In order to consider the influences of spatial distance and node degree on network growth, each new addition node judges degrees of existing nodes and their distances from itself, which become a basis to connect some existing nodes with certain probability. According to previous studies^17,19,22^ and practical experience, both the big node degree and short spatial distance contribute to the connection probability of two nodes. In other words, two closer nodes with each other are more likely to be connected, and nodes prefer to attach hubs. Thus, we must obey such rules during model. Specifically, for the interaction form of such two rules we are able to adopt the product form referring to literatures^17,31,32^.

The model is constructed in the following way:

1. Initial condition: We start with *m*_0_ all-to-all connected nodes on the geographical space.
2. Growth: At every time step, a new node is added, which is randomly placed on the geographical space.
3. Addition of edges: New node *j* connects *m* (*m* ≤ *m*_0_) existing nodes, which are selected with the probability

1 $${P}_{ij}=\frac{{k}_{i}^{\alpha }f({r}_{ij})}{\sum _{i < j}^{N}\,{k}_{i}^{\alpha }f({r}_{ij})},$$

where *k*_*i*_ is the degree of existing node *i*, *r*_*ij*_ is the spatial distance between them, $f({r}_{ij})=\frac{1}{\sqrt{2\pi }}\exp (-\frac{{r}_{ij}^{2}}{2})$. *α* is an independent and tunable system parameter, *N* is the total number of nodes.

The growing process of networks repeats step (2) and (3) until the network achieves the desired size. Accordingly, at each step, the number of nodes increases by one, while the number of edges increases by *m*. In Eq. (1), *α* can be various but greater than or equal to one referring to ref.^32^, which directly determine the attraction heterogeneity of nodes with different degrees and represent the competition extent between big hubs for new addition nodes during the growing process of networks. Furthermore, if *α* is large, there can be not only higher possibility to connect nearer nodes but also a little bigger chance for a hub to connect far nodes. For small values of *α*, the possibility for a hub to connect far nodes becomes small. However, *f*(*r*_*ij*_) reflects the effects of geographical distance on network growth, new addition node *i* prefers to connect existing neighbor nodes. Because of the shorter distance between new addition node *j* and existing node *i*, more possibly they are connected with each other. In our geographical model, the distance function *f*(*r*_*ij*_) is different from *r*^−*α*^ in ref.^32^ and ${e}^{-\lambda {r}_{ij}}$ in ref.^14^. Because there should exist an initial optimal area range for each object of real network in our heart, i.e., it is quite natural for people to tend to make friends with persons in the local area at the beginning though such range will be feeble in the future. Thus, $f({r}_{ij})=\frac{1}{\sqrt{2\pi }}\exp (-\frac{{r}_{ij}^{2}}{2})$ in our model. Obviously, geographical distances and node degrees together work, but interactions between top hubs must complicate the growing process of network, and lead to interesting phenomena.

## Simulations and Analysis

In this section, we will give the degree distributions and spatial distributions of top hubs and mainly explore the underlying mechanisms of different distribution features of top four hubs. Then, we would uncover the promotion effect of different geographical areas on the growth of top hubs through the average degrees at different spatial positions, and investigate the relationships between position and degree distributions for top hubs.

### Degree distributions of top hubs

**Figure 1:**
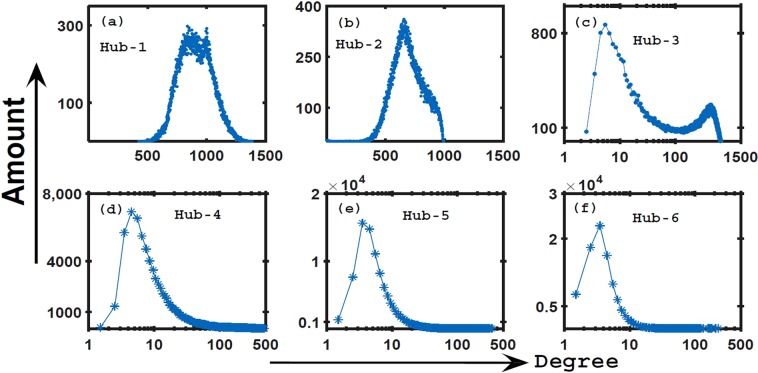
The degree distributions of top six hubs. (**a**–**e**) Respectively correspond to Hub-1 ~ Hub-6, where the spatial size *L* = 10 and *m*_0_ = 2, *m* = 1. Such distributions origin from 10^5^ geographical networks.

In order to investigate the statistical features of degree distributions of different big hubs in geographical networks, we use Eq. (1) to construct 10^5^ networks with *α* = 2.0 and *N* = 2000. The degree distributions of top six hubs are shown in Fig. 1. It is obvious that the degree distributions of top four hubs are very different. It is a distribution of single peak for Hub-1. Moreover, there is a wrinkle on the peak. Although the degree distribution of Hub-2 is also a single peak, there exist an obvious degree limit, 1000. Differently from Hub-1 and -2, Hub-3 is a distribution of double peaks. For Hub-4, 5 and 6, their degree values concentrate on a small value, less than 10. Furthermore, Hub-4’s distribution is similar with Hub-5 and Hub-6. In fact, we have compared Hub-4 with other smaller hubs, they also have similar distribution features. Therefore, we mainly analyze the top four hubs in the following explorations to find out the mechanism of such different degree distributions in geographical spaces.

**Figure 2:**
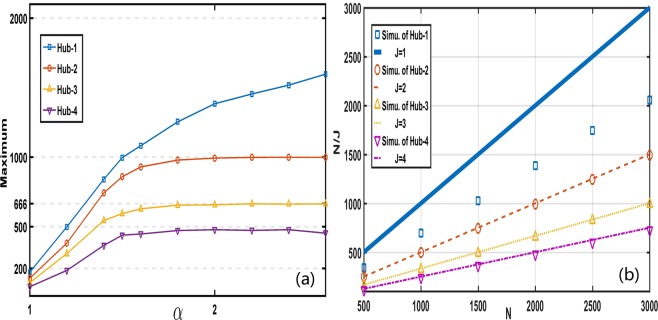
The maximal degrees and upper limits of different top hubs vary with *α* and network size *N*, the related parameters are the same as Fig. 1. (**a**) The maximal degrees of different top hubs vary with *α*. The maximums of hubs grow with *α* increasing, and at last arrive at upper limits. (**b**) The variations of the upper limits of top hubs respectively from numerical simulations and the predictions of $\frac{N}{J}$ with network size *N* when *α* = 2.0, where square, circle, up triangle and down triangle lines (bold solid, dash, dot and dash-dot lines) respectively correspond to Hub-1 ~ Hub-4 from the simulation results (the predictions of $\frac{N}{J}$).

According to Eq. (1), the variation of *α* reflects the intensity of preferential attachment of degree. Therefore, such increasing intensity of preferential attachment can promote the degrees of hubs. In Fig. 2(a), we give the maximal degrees of Hub-2 under various *α* through the great number of network samples. It is apparent that there exist upper limit. According to Eq. (1), when *α* becomes large, the degree advantage of hubs is amplified and make hubs more attractive for new addition nodes, so we can see that the maximum of Hub-2 grows with *α* when *α* is small (around *α* = 1), as shown in Fig. 2(a). However, it is impossible to grow forever. The maximum of Hub-2 is impossible to be larger than Hub-1, the most ideal situation for Hub-2 is that Hub-1 and Hub-2 have the same size and together increase to upper limit. Therefore, it is easy to be inferred that the upper limit of Hub-2 is *N*/2. In Fig. 1, *N* = 2000, so the upper limit of Hub-2 is *N*/2 = 1000, which is the reason that the degree values of Hub-2 are all less than 1000 in Fig. 1(b), and also leads to the plat of Hub-2 in Fig. 2(a) when *α* is large enough (e.g. around *α* = 2.0). According to the above analysis, we can infer the upper limit of each top hub, $\frac{N}{J}$, where *N* and *J* are respectively the number of network nodes and the rank of top hub. In Fig. 2(a), simulations from lots of geographical network samples testify the prediction results of $\frac{N}{J}$ for top four hubs. At the same time, in Fig. 2(b) we give the variations of upper limits of top hubs with the size of network *N*. Excitingly, the numerical results and the predictions of $\frac{N}{J}$ coincide again, which together suggest that bigger networks correspond to bigger upper limits for every top hub. Usually, when *α* is not large enough, Hub-1 can not defeat all other hubs and realize nearly all node connections. Thus, we have not see the upper limit of Hub-1 from simulations reach *N* in both Fig. 2(a,b), no matter how big network size is.

**Figure 3:**
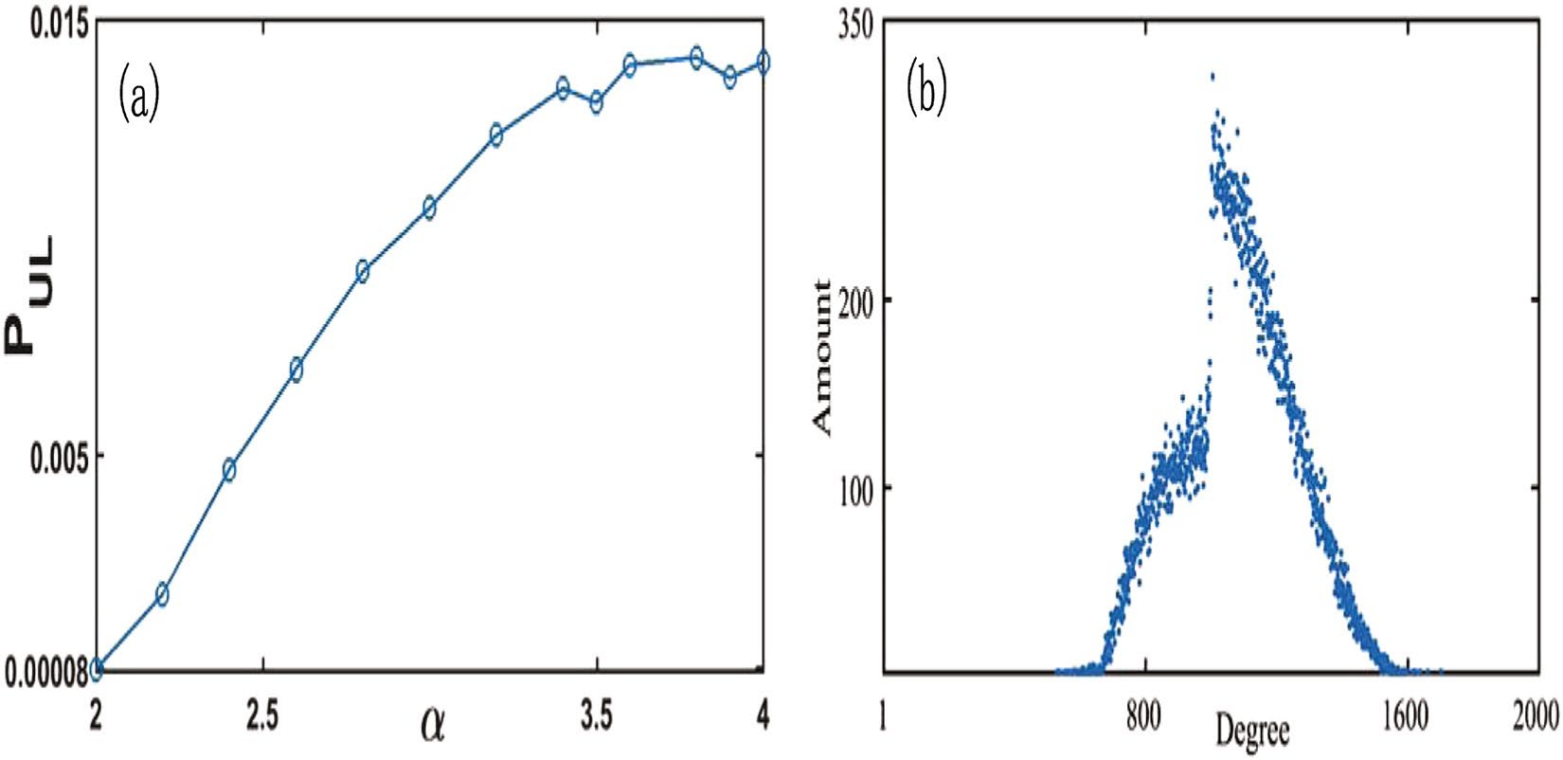
(**a**) The probability of Hub-2 with the degree larger than 990 over *α*, and each *P*_*UL*_ is from 10^5^ example networks. (**b**) The degree distribution of Hub-1 at *α* = 2.8 from 10^5^ example networks. Both in (**a**) and (**b**) with *N* = 2000.

When *α* grows, the attraction ability of Hub-2 and Hub-1 are all strengthened continuously, which result the probability around the maximal degree grows for Hub-2, as shown in Fig. 3(a). In fact, that is the reason of the right abnormal distribution (i.e. asymmetric distribution peak) of Hub-2 in Fig. 1(b). Meaningly, the growth of *α* can both improve attraction ability of hubs to new nodes and result in the severer competitions between hubs. With the probability of degree around *N*/2 increasing for Hub-2 (as shown in Fig. 3(a)), the probability less than *N*/2 for Hub-1 must be small because of the extrusion of Hub-2 and the growth of own attraction for new addition nodes, and the distribution larger than *N*/2 would increase for Hub-1. Therefore, there exist a wrinkle for Hub-1 at *α* = 2.0 in Fig. 1(a). If *α* could be bigger, the wrinkle of Hub-1 in Fig. 1(a) turn to be a scar, as shown in Fig. 3(b), where the distribution of degree larger than *N*/2 for Hub-1 is much stronger than the one smaller than *N*/2. Meanwhile, the probability around *N*/2 for Hub-2 is much more than *α* = 2.0, as shown in Fig. 3(a).

**Figure 4:**
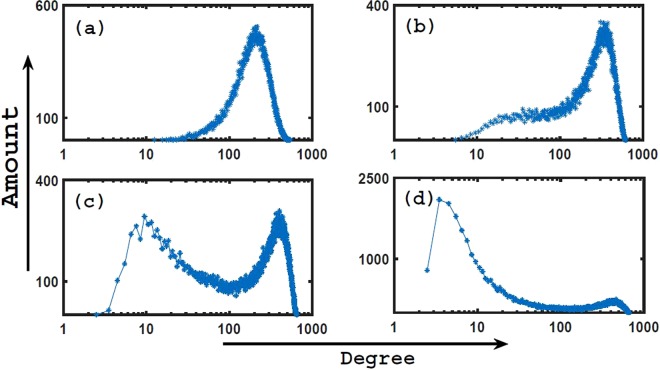
The degree distributions of Hub-3. (**a**–**d**) Respectively correspond to *α* = 1.4, 1.6, 1.8 and 2.2. Each distribution are from 10^5^ example networks with *N* = 2000. Bigger *α* means that top two hubs with stronger attraction more severely squeeze Hub-3.

Actually, with the growth of *α* the attraction ability of each hub for new addition nodes are all strengthened, which lead to the maximal degree of each top hub become bigger in the statistics, as shown in Fig. 2(a). Accordingly, the competitions between hubs are also intensified. When *α* is small (e.g., closer to 1), the attraction of top hub is not enough to overcome geographical measure to get great degree and competition between top three hubs is weak because of geometrical measure, the degrees of different top hubs easily resemble each other from Fig. 2(a). Consequently, Hub-3 can tend to take a degree distribution of single peak, as shown in Fig. 4(a). However, when *α* gradually becomes large, Hub-1 and Hub-2, as top two hubs, begin to use the advantage of degree values to get enough strong attraction for new addition nodes, which may break the constraint of spatial distance between nodes, and simultaneously intensify competitions between top hubs. Such situations cause Hub-3 difficult to get a big degree and more possible to be a small hub. So the low-degree distribution of Hub-3 is enhanced. That is the reason of the difference between Fig. 4(b) (the degree distribution of Hub-3 for *α* = 1.6) and Fig. 4(a) (*α* = 1.4). With the further growth of *α*, Hub-1 and Hub-2 depend on degree advantage to severely inhibit other hubs like Hub-3 to make other hubs small in more possibility, which lead to the double peak degree distribution of Hub-3 in Fig. 4(c). Thus, two peaks of Hub-3 in Fig. 4(c), one concentrates on small degree values, the other concentrates on big ones. However, if *α* becomes too big, Hub-1 and Hub-2 excessively squeeze Hub-3 and make the possibility of Hub-3 with big degree very low, but the possibility of small degree rapidly rises. Such situation can be seen in Fig. 1(c) (*α* = 2.0) and Fig. 4(d) (*α* = 2.2), where the right distribution peak have become much lower than the left for Hub-3. The above sight is the origin of the degree distribution of Fig. 1(c) for Hub-3.

In fact, the increasing extrusion that Hub-3 faces during the growth of *α*, also exists for Hub-4 and other smaller hubs. Unluckily, Hub-4 and smaller hubs are much more fragile than Hub-3. Therefore, when *α* grows, the degree distribution of Hub-4 *et al*. have no chance to appear double peak distribution like Hub-3, directly take on single peak and concentrate a small value. Apparently, Hub-4 and other smaller hubs face the predicament of Hub-3 but get worse results about degree values because of the lower competitiveness. So we can see that the distributions of degree values from Hub-4 to Hub-6 are very similar in Fig. 1, and their degree distributions mainly concentrate on very small values (≤10).

Generally, growing *α* leads to that big hubs get stronger degree attraction, which is good for being bigger hub. Interestingly, growing *α* also fires competitions of different top hubs because the enough large attraction could cross spatial distance and generate extrusion, which directly causes the differences of degree distributions for different hubs, such as the wrinkle for Hub-1 in Fig. 1(a), the distribution of two peaks for Hub-3 in Fig. 1(c) and so on.

### Features of spatial distribution

**Figure 5:**
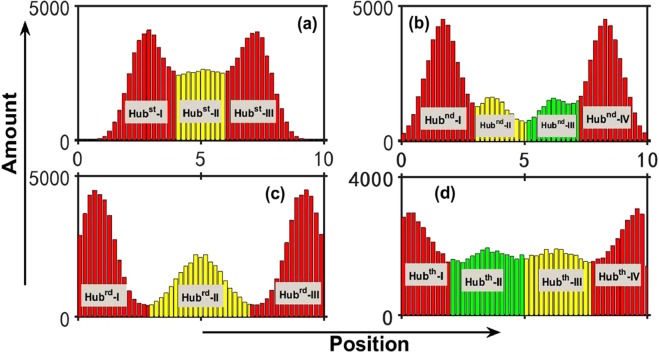
(**a**–**d**) Are respectively the spatial distributions of Hub-1 ~ Hub-4 from Eq. (1), where *α* = 2.0, *L* = 10 and each distribution are from 10^5^ example networks. According to distribution peaks, each distribution is divided into several distribution areas. For the same hub, different distribution areas are indexed by different colors and Roman numerals.

**Figure 6:**
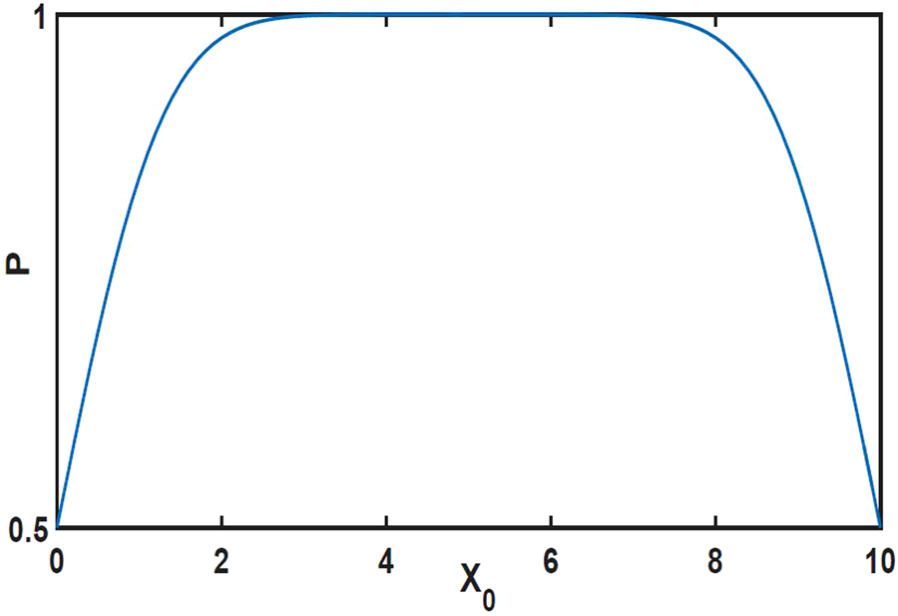
The extent of easiness for nodes at different positions to connect others in the whole spatial space *L*. The positions at the plat of *P*(*x*_0_) are almost equivalent. The positions near the spatial boundaries are bad for big degree generation.

According to the above analysis, the degree distributions of top hubs are diverse in geographical networks. Actually, the spatial distribution of top hubs are also different from each other. In Fig. 5 we give the spatial distributions of top four hubs with *α* = 2.0. Such four distributions are very different from each other though they are all distributions with multiple peaks. For instance, the number of distribution peaks for Hub-3 in Fig. 5(c) is not the same as Hub-2 in Fig. 5(b). Moreover, the distance of distribution peaks from spatial boundary are different for each top hub. For easy explorations we have divided the spatial distribution of each hub into several parts on basis of distribution peaks and indexed them, as shown in Fig. 5. When we check distribution areas of different top hubs in geographical networks, it is natural to ask the mechanism of such spatial distributions. The growing process of geographical networks is governed by Eq. (1). According to the factor $f(\,\cdot \,)$, if we define $P({x}_{0})={\int }_{L}\,f(x-{x}_{0})dx$, *x*_0_ and *x* are the position of existing node and new addition node, respectively. *P*(*x*_0_) implies the extent of easiness for a node at *x*_0_ to connect other nodes in the whole space. As shown in Fig. 6, *P* is various for different *x*_0_. There exist a plat for *P* in the middle part of geographical space, where *x*_0_ is almost equivalent. But *P* is small near spatial boundaries, which suggest that the attraction ability of nodes there are relatively weak. The advantage at plat area in Fig. 6 is easier for nodes to connect new addition nodes, obviously, which is good for realizing big degree values. Naturally, for Hub-1, as the biggest hub, there should have existed the highest distribution peaks at the middle of plat area. However, the fact is that the two symmetric high distribution peaks (*Hub*^*st*^-*I* and *Hub*^*st*^-*III*) locate at the two sides of the spatial centre, while the spatial centre have low distribution probability for Hub-1 from Fig. 5(a) (i.e. *Hub*^*st*^-*II*). As we know, the advantage of plat area is suitable for any nodes, so all top hubs prefer to gather there during the process of network growth, which lead to much more intensive competitions than other places. As the biggest hub, Hub-1 would suffer great extrusions from all other hubs and need subdue the more obstructive provided it arrive at the middle. So *Hub*^*st*^-*II* area of Hub-1 at the middle part of spatial space are lower than *Hub*^*st*^-*I* and *Hub*^*st*^-*III* in Fig. 5(a). Compared with the middle *Hub*^*st*^-*II* area, the two symmetric peaks (*Hub*^*st*^-*I* and *Hub*^*st*^-*III*) in Fig. 5(a) are better for big degree, which both evade much more intensive competitions and receive the distance advantage from nodes around spatial boundaries, after all, there is marginal difference for different *x*_0_ at the plat area of Fig. 6. In fact, the above effects are both the source of the symmetrical peaks of other hubs, such as *Hub*^*nd*^-*I* (*Hub*^*nd*^-*II*) and *Hub*^*nd*^-*IV* (*Hub*^*nd*^-*III*) in Fig. 5(b), *Hub*^*rd*^-*I* and *Hub*^*rd*^-*III* in Fig. 5(c), and *Hub*^*th*^-*I* (*Hub*^*th*^-*II*) and *Hub*^*th*^-*IV* (*Hub*^*th*^-*III*) in Fig. 5(d), and the reason that the distribution of each hub at the middle part of geographical space are lower. On the other hand, top hubs can not be too close with spatial boundaries (for instance, *Hub*^*st*^-*I*, *Hub*^*nd*^-*IV* and so on), which makes itself difficult to connect many other nodes according to Fig. 6 and easy to reduce degree. Unfortunately, Hub-4 under the extrusion of top three hubs are much closer with spatial boundary (such as *Hub*^*th*^-*I* and *Hub*^*th*^-*IV*) from Fig. 5(d), which is the reason of being a joey compared to the top three hubs in Fig. 1.

Obviously, according to Fig. 5, there exist multiple peaks in spatial distribution for each top hub, and their statistical distribution areas overlap. But such overlaps do not mean that top hubs can together appear at the same position. Actually, there exist clear corresponding relationships for spatial distribution peaks of different top hubs in the same geographical network. In Table 1, we give the corresponding relationships. According to Table 1, there totally exist 24 kinds of corresponding relationships on basis of spatial distribution areas in Fig. 5 for top four hubs. Apparently, Hub-1 and Hub-2 tend to face each other across the spatial centre, i.e. Hub^*st*^-I (Hub^*st*^-III) and Hub^*nd*^-IV (Hub^*nd*^-I) respectively locate at the two sides of spatial centre (e.g., Nos 9 and 10 in Table 1), which indicates that there exist repulsion between such top two hubs because of intensive competition, and only apart far away to make them healthfully grow when network grow. Even though Hub-1 stay at the middle part of space (Hub^*s*^*t*-II), Hub-2 can also select Hub^*nd*^-I or -IV (such as Nos 8 and 17 in Table 1) and try to avoid the distribution overlap. So top hub competitions can be glimpsed from Hub-1 and Hub-2. Hub-3 is smaller than Hub-2, which make the attraction ability of Hub-3 to new addition nodes weaker than Hub-1 and Hub-2. So the spatial distribution peaks of Hub-3 always locate at the remaining gaps (such as Nos 2, 3, 4 and 5 in Table 1) except Hub-1 and Hub-2. In top four hubs, Hub-4 is the smallest, and the degree is mainly distributed around 10 from Fig. 1, which make Hub-4 almost robust towards different corresponding relationships of top three hubs. Any gap between top three hubs can generate little Hub-4. Therefore, each distribution area of Hub-4 can always match different corresponding relationships of top three hubs, as shown in Table 1. Table 1 should have included smaller hubs than Hub-4, because the degree of other hubs are too small and the spatial distributions are more common than Hub-4, and there exist few outstanding features. As a result, Table 1 only include top four hubs.

**Table 1:** The statistics of corresponding relationships of different distribution areas for top four hubs.

1	Hub^*st*^-III	Hub^*nd*^-II	Hub^*rd*^-I	Hub^*th*^-I
2	Hub^*st*^-I ˄ Hub^*st*^-II	Hub^*nd*^-IV	Hub^*rd*^-I	Hub^*th*^-I
3	Hub^*st*^-III	Hub^*nd*^-I	Hub^*rd*^-II	Hub^*th*^-I
4	Hub^*st*^-I	Hub^*nd*^-IV	Hub^*rd*^-II	Hub^*th*^-I
5	Hub^*st*^-II ˄ Hub^*st*^-III	Hub^*nd*^-I	Hub^*rd*^-III	Hub^*th*^-I
6	Hub^*st*^-I	Hub^*nd*^-III	Hub^*rd*^-III	Hub^*th*^-I
7	Hub^*st*^-III	Hub^*nd*^-II	Hub^*rd*^-I	Hub^*th*^-II
8	Hub^*st*^-II	Hub^*nd*^-IV	Hub^*rd*^-I	Hub^*th*^-II
9	Hub^*st*^-III	Hub^*nd*^-I	Hub^*rd*^-II	Hub^*th*^-II
10	Hub^*st*^-I	Hub^*nd*^-IV	Hub^*rd*^-II	Hub^*th*^-II
11	Hub^*st*^-II ˄ Hub^*st*^-III	Hub^*nd*^-I	Hub^*rd*^-III	Hub^*th*^-II
12	Hub^*st*^-I	Hub^*nd*^-III	Hub^*rd*^-III	Hub^*th*^-II
13	Hub^*st*^-III	Hub^*nd*^-II	Hub^*rd*^-I	Hub^*th*^-III
14	Hub^*st*^-I ˄ Hub^*st*^-II	Hub^*nd*^-IV	Hub^*rd*^-I	Hub^*th*^-III
15	Hub^*st*^-III	Hub^*nd*^-I	Hub^*rd*^-II	Hub^*th*^-III
16	Hub^*st*^-I	Hub^*nd*^-IV	Hub^*rd*^-II	Hub^*th*^-III
17	Hub^*st*^-II	Hub^*nd*^-I	Hub^*rd*^-III	Hub^*th*^-III
18	Hub^*st*^-I	Hub^*nd*^-III	Hub^*rd*^-III	Hub^*th*^-III
19	Hub^*st*^-III	Hub^*nd*^-II	Hub^*rd*^-I	Hub^*th*^-IV
20	Hub^*st*^-I ˄ Hub^*st*^-II	Hub^*nd*^-IV	Hub^*rd*^-I	Hub^*th*^-IV
21	Hub^*st*^-III	Hub^*nd*^-I	Hub^*rd*^-II	Hub^*th*^-IV
22	Hub^*st*^-I	Hub^*nd*^-IV	Hub^*rd*^-II	Hub^*th*^-IV
23	Hub^*st*^-II ˄ Hub^*st*^-III	Hub^*nd*^-I	Hub^*rd*^-III	Hub^*th*^-IV
24	Hub^*st*^-I	Hub^*nd*^-III	Hub^*rd*^-III	Hub^*th*^-IV

The area indexes are the same as Fig. 5, the symbol “˄” represent the junction of two distribution areas. There exist 24 corresponding relationships, which suggest top four hubs can not locate the same position in the same geographical network.

According to Table 1, it is easy to understand the small distribution peaks of top hubs. For instance, due to No. 6 in Table 1, Hub-2 stays at Hub^*nd*^-III when Hub-1’s stays at Hub^*st*^-I. Such condition lead to the distance between Hub-2 and Hub-1 is too close, which make Hub-2 suffers severe inhibition from Hub-1 during growth. As a result, the distribution possibility at Hub^*nd*^-III for Hub-2 is low, the corresponding distribution peak here is low. Luckily, Hub^*rd*^-III and Hub^*th*^-I become much good respectively for Hub-3 and Hub-4, so there exist two high distribution peaks. Meaningly, the corresponding relationships of hub distributions in Table 1 uncover the underlying mechanism of different hub distributions in Fig. 5, imply the origin of diversity of the spatial distribution peaks for the same top hub, and suggest the evolution diversity of growing geographical networks. In the real geographical networks, the actual result may be one of our multiple corresponding distributions in Table 1 because of the influences of other complicated reasons.

### The features of average degrees

**Figure 7:**
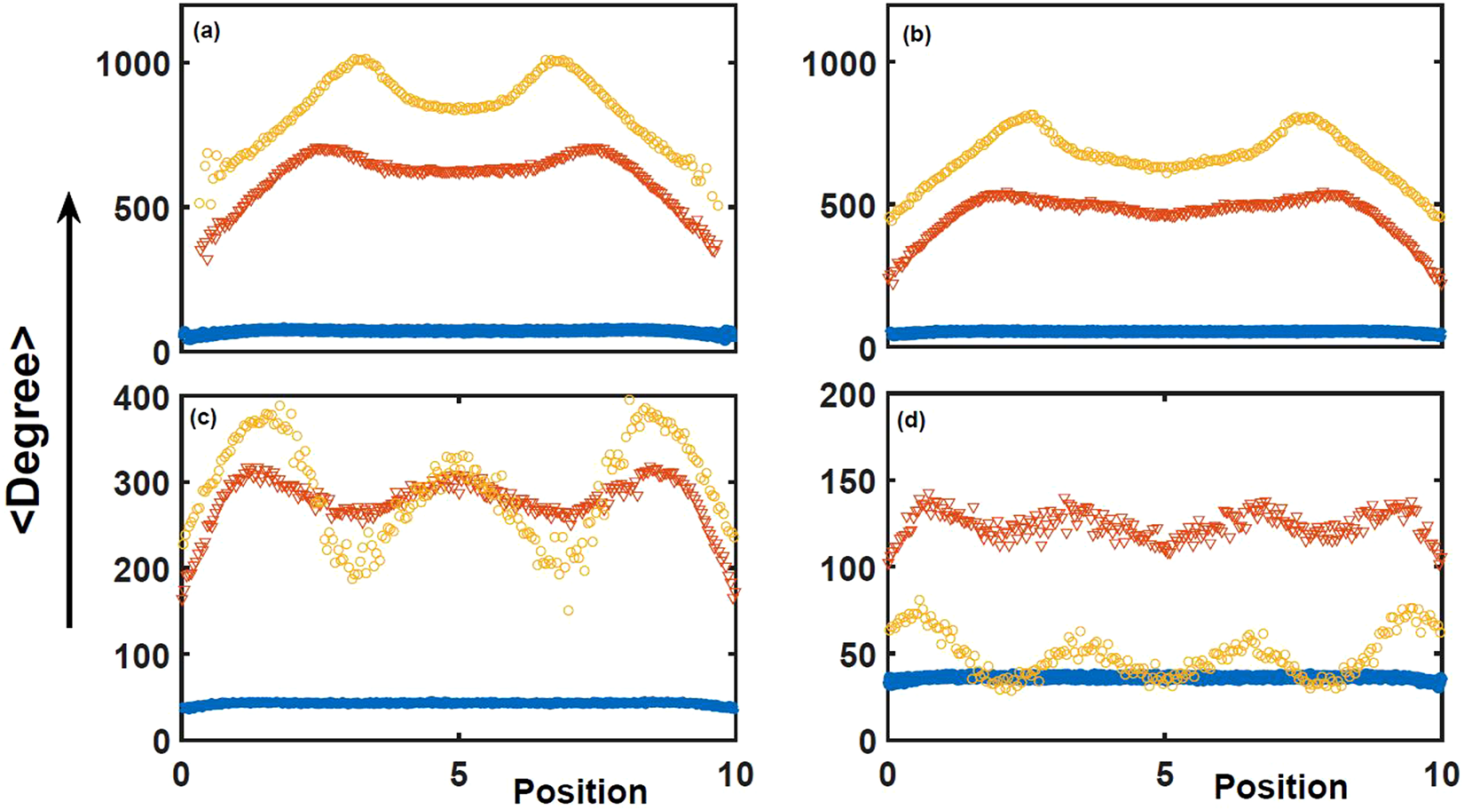
The average degree over different spatial positions. (**a**–**d**) Respectively correspond to Hub-1 ~ Hub-4 at different *α*, where *α* = 1.0, 1.5, 2.0 respectively for star lines, triangle lines and circle lines. The average degrees at different positions for Hub-1 and Hub-2 grow with *α*, while for Hub-3 and Hub-4 they rise first, then descend.

During the growth of geographical networks, the degree and geographical measures together influence the possibility of a node to become a big hub according to Eq. (1). In Fig. 7, the average degrees over different positions are given for top four hubs. When *α* = 1.0 (star lines in Fig. 7) increases to *α* = 1.5 (triangle lines in Fig. 7), the average degrees become bigger for each position of each top hub in geographical space, which means that each top hub gains stronger attraction and has big degree. Interestingly, during such process, the average degrees at different positions appear obvious differences for each top hub. For instance, the average degree of Hub-3 show three peaks at different positions in Fig. 7(c), and the average degrees near geographical boundary are very small. It is natural for us to know the geographical boundary is bad for growth of top hubs from Fig. 6. Actually, the emergent variations of spatial distribution for the top hub in Fig. 5 just suggest the diverse effects of different spatial positions on the growth of top hub. Some areas are fertile soil, others are poor. It is worthwhile to note that something poor or fertile is close related to growth situation. When *α* = 1.0, the average degree (the star lines in Fig. 7) are almost equivalent at each position and we can not see the outstanding diverse effects of spatial positions on each top hub. But once *α* becomes large (*α* = 1.5 in Fig. 7), the spatial differences emerge (the triangle lines in Fig. 7). In other words, the advantages of geographical positions must match the attraction ability of top hubs. The attraction is too weak (e.g. *α* = 1) to enjoy position benefits. For the same hub, the bigger *α* can uncover the more outstanding difference of geographical positions, as is shown in the triangle lines in Fig. 7. If the circle lines in Fig. 7 (*α* = 2.0) are compared to Fig. 5, it is easy to find the geographical positions for bigger average degrees are just the spatial distribution peaks in Fig. 5, which means that such geographical areas promoting degrees are just where top hubs favor to appear more often. The distribution peaks of top hubs must locate the fertile soil. In fact, although the growth of *α* is good for the attraction ability and the position advantage is good for big degree, too large *α* (e.g. *α* = 2.0) is no longer satisfactory for all top hubs. In Fig. 7, the circle lines show that the average degree become bigger and the spatial difference are more outstanding for Hub-1 and -2, but the average degree of Hub-4 suddenly falls at each position (circle lines in Fig. 7(d)), whose spatial differences decrease. Significantly, the average degree at each spatial position are not all bigger from *α* = 1.5 to *α* = 2.0 for Hub-3, as shown in circle lines in Fig. 7(c). Actually, the peculiar cases from Hub-3 and -4 suggest that too large *α* lead to the excessive attraction of top two hubs, which make competitions between top hubs intense and seriously squeeze the living space of Hub-4. Therefore, the average degree of Hub-4 suddenly become small for *α* = 2.0. Meanwhile, Hub-3 is also battered. However, the attraction of Hub-3 is better than Hub-4, Hub-3 does not respond sharply, after all, there exist falling average degree in partial areas from Fig. 7(c). Accordingly, if we continue to increase *α*, the average degree of Hub-3 would fall like Hub-4. For another hand, the two peaks of Hub-1 in Fig. 7(a) take on the trend towards the spatial center with *α* increasing from 1.5 to 2.0, which further verify the above discussion, it needs more powerful attraction for Hub-1 to possess the middle position of geographical space.

Generally, the geographical effect should be coupled with the enough attraction of hubs, which makes each geographical position play constructive and different roles. Neither too strong nor weak attraction ability are good for geographical space roles, even for individual top hubs. Correspondingly, in real systems it is necessary to guarantee that different factors work appropriately, any too strong factor may lead to peculiar results.

## Summary

In this paper, we propose a growing network model embedded into geographical space, and mainly explore the spatial distributions and degree distributions of top hubs. We find that the degree distributions of top four hubs are different from each other. The differences of degree distributions result from specific attraction powers and competition intensity between top hubs during the growth process in geographical space. Such situations seriously depend on tunable parameter *α*, which could work together with spatial positions of top hubs to influence the degree distribution of top hubs. Meanwhile, the spatial distributions of top four hubs are diverse, though they are all multiple peaks. Different spatial positions suit for the growth of different sizes of top hubs, the competitions and repulsion effects between top hubs make ranks of top hubs and generate clear corresponding relationships of spatial distribution areas. Through the average degrees at different spatial positions for top hubs, we find the peaks of spatial distribution areas for top hubs are just the hotbed of big degree. Furthermore, the excessive competition also make top hubs of low ranks be squeezed and battered (such as Hub-3 and -4). Significantly, the competition in this paper are mainly from degree advantage (meaning attraction ability), and the geographical condition play aided function. As a result, the advantages of spatial positions play important role just when top hubs have enough attraction. In addition, our model are different from gravity models in literatures^23^, but our results suggest the similar conclusion that the geographical effect are auxiliary or second.

Growing networks embedded in geographical space are a class of important networks. The related studies may offer real systems new inspirations about the real functions of top hubs from degree distributions and spatial distributions. In ref.^33^, Pan *et al*. used the trade network data from 29 provinces in China from 2006 to 2011 to explore the network hubs, which indicate that hubs are unevenly distributed in China. Such feature is similar with the heterogeneous spatial distribution of top hubs in this paper. And it is a much desirable case that more and more real data take on similar or even same conclusions with ours in future. We hope our related results may be helpful for understanding the features of top hubs in real networks. On the basis of the present results, we will further consider other related features of geographical networks in future.
